# A novel model for predicting intravenous immunoglobulin-resistance in Kawasaki disease: a large cohort study

**DOI:** 10.3389/fcvm.2023.1226592

**Published:** 2023-07-28

**Authors:** Shuhui Wang, Chuxin Ding, Qiyue Zhang, Miao Hou, Ye Chen, Hongbiao Huang, Guanghui Qian, Daoping Yang, Changqing Tang, Yiming Zheng, Li Huang, Lei Xu, Jiaying Zhang, Yang Gao, Wenyu Zhuo, Bihe Zeng, Haitao Lv

**Affiliations:** ^1^ Department of Cardiology, Children’s Hospital of Soochow University, Suzhou, China; ^2^ Department of Pediatrics, Institute of Pediatric Research, Children's Hospital of Soochow University, Suzhou, China

**Keywords:** Kawasaki disease, intravenous immunoglobulin resistance, prediction model, C-reactive protein to albumin ratio (CAR), children

## Abstract

**Background:**

Predicting intravenous immunoglobulin (IVIG)-resistant Kawasaki disease (KD) can aid early treatment and prevent coronary artery lesions. A clinically consistent predictive model was developed for IVIG resistance in KD.

**Methods:**

In this retrospective cohort study of children diagnosed with KD from January 1, 2016 to December 31, 2021, a scoring system was constructed. A prospective model validation was performed using the dataset of children with KD diagnosed from January 1 to June 2022. The least absolute shrinkage and selection operator (LASSO) regression analysis optimally selected baseline variables. Multivariate logistic regression incorporated predictors from the LASSO regression analysis to construct the model. Using selected variables, a nomogram was developed. The calibration plot, area under the receiver operating characteristic curve (AUC), and clinical impact curve (CIC) were used to evaluate model performance.

**Results:**

Of 1975, 1,259 children (1,177 IVIG-sensitive and 82 IVIG-resistant KD) were included in the training set. Lymphocyte percentage; C-reactive protein/albumin ratio (CAR); and aspartate aminotransferase, sodium, and total bilirubin levels, were risk factors for IVIG resistance. The training set AUC was 0.825 (sensitivity, 0.723; specificity, 0.744). CIC indicated good clinical application of the nomogram.

**Conclusion:**

The nomogram can well predict IVIG resistance in KD. CAR was an important marker in predicting IVIG resistance in Kawasaki disease.

## Introduction

Kawasaki disease (KD), an acute systemic vasculitis, involves the coronary artery that occurs in children less than 5 years of age and is the main reason for acquired heart disease in children (1). Coronary artery lesions (CALs) are the most common and life-threatening complications of KD. If not treated in time, CALs can lead to coronary artery dilatation, coronary artery aneurysm formation that lead to long-term sequelae such as lumen stenosis or occlusion, and in severe cases, myocardial infarction, which is life-threatening (2). High-dose intravenous immunoglobulin (IVIG) (2 g/kg) combined with aspirin is the first-line treatment of KD; this treatment reduces the incidence of CALs from 20%–25% to 2%–4% (1). However, initial IVIG treatment is not effective in 7.5%–26.8% of KD children who continue to experience inflammatory reaction, and these patients remain at risk for developing CALs. Moreover, severe complications such as Kawasaki disease shock syndrome or macrophage activation syndrome may occur and can be life-threatening (3). Therefore, an in-depth analysis of the factors influencing IVIG insensitivity in KD, early prediction, and timely and effective intervention, are urgent clinical requirements to reasonably reduce the risk of cardiac damage in IVIG-resistant KD.

Clinical prediction models are quantitative tools for risk and benefit assessment and provide intuitive and rational information to physicians, patients, and medical policy makers in the early stage of disease development. Predictive models have been recommended for clinical risk assessment (4). At present, researchers in many countries and regions have established region-specific predictive scoring systems based on the clinical data of children with KD. However, owing to the small sample size in these studies, the choice of modeling methods, and the lack of internal and external validation, the sensitivity and specificity of prediction models either do not meet clinical expectations or cannot be applied in different populations (5–9). Therefore, establishing a scoring model with a large sample size, reasonable modeling approach and strong predictive ability based on internal and external validation is essential.

KD involves the release of many inflammatory factors and the cascade amplification effect. Previous studies have used platelet count, C-reactive protein (CRP), and neutrophil measurements (peripheral neutrophil count and neutrophil%), and serum albumin level to predict IVIG resistance (10–12). Several studies have shown that a combination of indicators, including the C-reactive protein to albumin ratio (CAR) (13), neutrophil to lymphocyte count (NLR) (14), prognostic nutritional index (15), and systemic immune-inflammation index (SII) (16) have better predictive performance in IVIG sensitivity and coronary artery injury, but these factors have not been tested in a prediction tool of IVIG resistance. Therefore, the aim of this study was to collect and analyze clinical data and combining indicators of KD, and to develop a new predictive scoring model for IVIG-resistant KD to facilitate early assessment and treatment.

## Patients and methods

### Subjects

**Figure 1:**
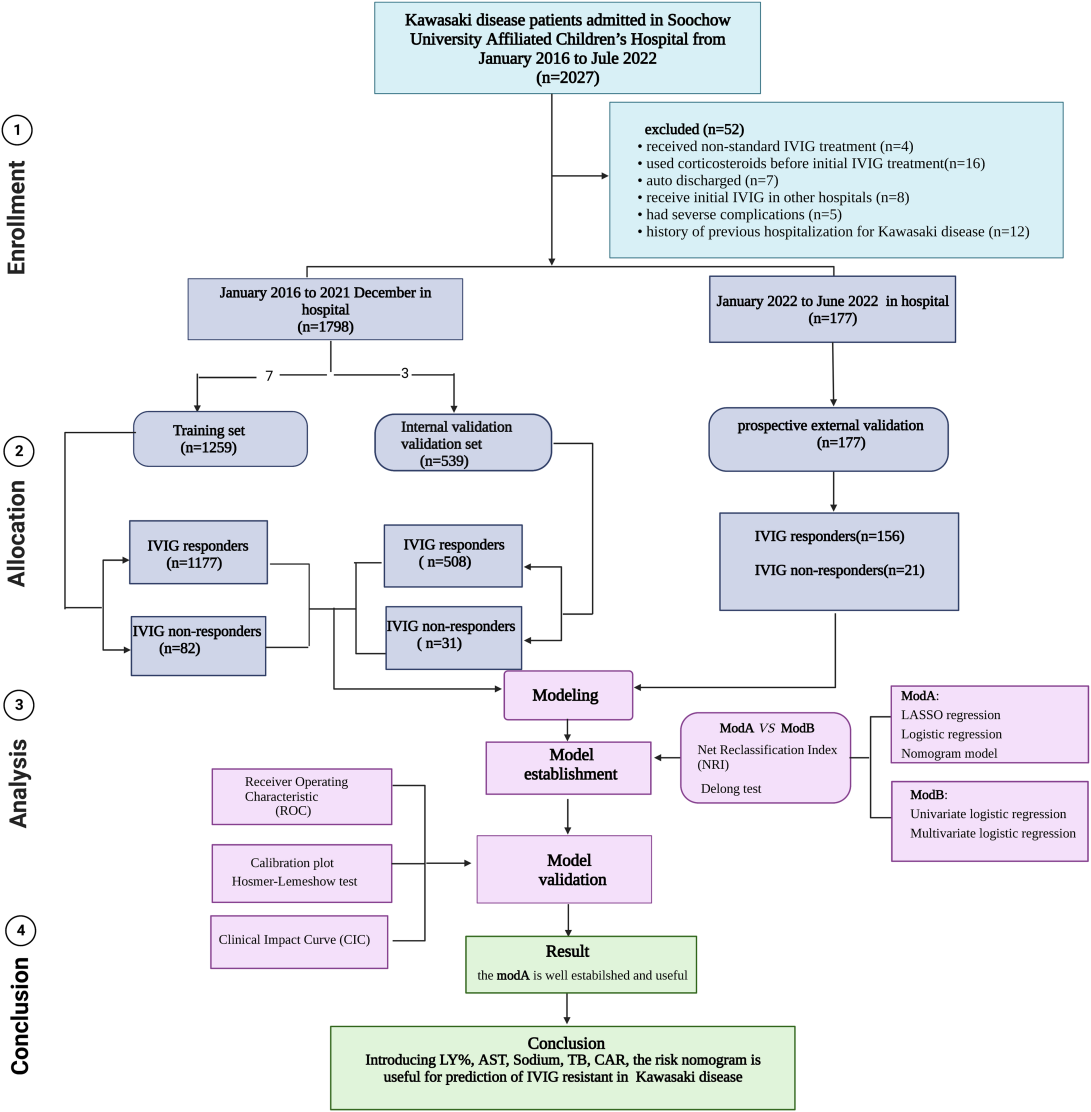
Flow diagram of prediction models for IVIG-resistant Kawasaki disease. IVIG: intravenous immunoglobulin.

The clinical data for children with KD in the Children's Hospital of Soochow University diagnosed from January 1, 2016 to December 31, 2021 were collected. Patients who were diagnosed as having complete or incomplete KD according to American Heart Association (AHA) criteria were eligible (1). Exclusion criteria: (1) KD children in whom glucocorticoids, immunosuppressants, or blood products were used within 1 month before the onset of KD; (2) no IVIG treatment or non-standard dose; (3) severe immune diseases such as immunodeficiency or chromosomal abnormalities; (4) children discharged automatically; and (5) those with a history of previous hospitalization for KD. The first day of fever was defined as the first day of illness. The study consisted of a training, internal validation, and prospective external validation sets. Children hospitalized at the same hospital from January 2016 to December 2021 were included into the training set (1,259 children) and internal validation (539 children) set based on a 7:3 ratio. The data of 177 children with KD hospitalized in the Children's Hospital of Soochow University from January 2022 to June 2022 were used for prospective external validation. A flow diagram of the study design is shown in Figure 1.

### Treatment

Febrile patients received a dose of 2 g/kg IVIG and 30–50 mg/kg/day aspirin. Aspirin dosage was reduced to 3–5 mg/kg/day until patients were afebrile for 3–4 days. Patients who were resistant to IVIG were treated with second dose of IVIG and (or) intravenous methylprednisolone. IVIG resistance was defined as persistent fever within 36 h after the first IVIG infusion was completed or fever recurrence after regression. A patient was considered afebrile when the body temperature was <37.5°C for more than 24 h (10).

Coronary artery disease was defined as *Z*-score ≥2 in the proximal and middle segments of the left main coronary artery (LMCA), the left anterior descending artery (LAD), the left circumflex coronary artery (LCX), and the right coronary artery (RCA). The *Z*-score was calculated according to the height, weight, and coronary artery diameter detected by echocardiography according to AHA guidelines (1). The study was approved by the Ethics Committee of Children's Hospital of Soochow University (approval No. 2023CS055) and performed in accordance with the Declaration of Helsinki. All parents of the study participants were aware of the study before enrollment and provided written informed consent for their children's participation in the study.

### Data collection

Variables included in the model included demographic characteristics (gender and age); clinical outcomes, and laboratory test results on the first day of admission, including CAL, CRP, white blood cell (WBC) count, neutrophil%, lymphocyte percentage (LY%), erythrocyte sedimentation rate, as well as levels of hemoglobin, platelet, total cholesterol, serum albumin (ALB), aspartate aminotransferase (AST), alanine aminotransferase (ALT), sodium, total bilirubin (TB). Calculations based on laboratory test results including CAR, bilirubin-to-albumin ratio, NLR, prognostic nutritional index (albumin [g/L] + 0.005 × lymphocyte counts [10^9^/L]), and SII (platelet count × [neutrophil count/lymphocyte count]). Color Doppler echocardiography was performed by two experienced pediatric echocardiographers, and the results of echocardiography were obtained by the cardiologist on the basis of the echocardiographic report.

### Clinical practicality analysis

A rationality analysis was conducted on the model to analyze the feasibility of predictive models in clinical settings.

### Statistical analyses

Statistical analyses were conducted using SPSS 24.0 (IBM Corp, Armonk, NY, USA) and R 4.2.0 (https://www.R-project.org). Measurement data were expressed as median (P25–P75), and the percentage of cases (%) was used to represent the count data. Chi-square test was used for categorical variables, two independent *t*-test was used for numerical variables conforming to normal distribution, and non-parametric Mann–Whitney *U*-test was used otherwise. First, the R *caret* package was used to randomly divide patients into a training set and a validation set for external validation that conformed to a theoretical ratio of 7:3, and then, the error in prediction of the quantitative response variables was minimized by constraining the model parameters using the least absolute shrinkage and selection operator (LASSO) regression analysis to reduce the regression coefficients of some variables to zero. Subsequently, a multivariable logistic regression analysis was used to construct a nomogram-based prediction model (ModA). The independent risk factors of IVIG resistance selected by univariate analysis were analyzed by multivariate logistic regression to establish a model (ModB). Multivariate logistic regression analysis was performed using stepwise logistic regression with backward stepwise regression. The DeLong test was used to compare the area under the curves (AUCs) of different prediction models and perform hypothesis tests. *p* < 0.05 was considered statistically significant. Area under the receiver operating characteristic (ROC) curve was calculated to evaluate the discrimination, while calibration curves and the Hosmer–Lemeshow test were to assess the calibration of the nomogram. We also used determined sensitivity and specificity, 95% confidence interval (CI), net reclassification index (NRI), clinical impact curve (CIC) to evaluate the model performance in the training set. The data from January 2022 to June 2022 from the Children's Hospital of Soochow University were used for external validation.

## Results

### Characteristics of the study cohort

Of the 2,027 patients with KD, the following were excluded: four patients who received non-standard IVIG treatment, 16 patients who were administered corticosteroids before initial IVIG treatment, seven patients who were auto discharged, eight patients who received initial IVIG treatment in other hospitals, five cases who had severe complications, and 12 patients who had a history of previous hospitalization for KD. Data for 1,259 patients were included into the training set and those for 539 patients were included in the validation set for internal validation, conforming to the theoretical ratio of 7:3. 113 (6.3%) cases were defined as IVIG non-responders. A total of 177 patients were enrolled from January to June 2022 for external validation, and the incidence of IVIG insensitivity was 11.9%. The predictive variables of the patients in the two sets are given in Table 1.

**Table 1:** Comparison of variables between IVIG responders and IVIG non-responders.

Variables	Training cohort (*n *=* *1,259)		Validation cohort (*n *=* *681)			
	Model development		Internal validation (n=539)		External validation (n=177)	
	IVIG responders (*n *=* *1,177)	IVIG non-responders (*n *=* *82)	IVIG responders (*n *=* *508)	IVIG non-responders (*n *=* *31)	IVIG responders (*n *=* *156)	IVIG non-responders (*n *=* *21)
Male, *n* (%)	690 (58.6%)	50 (61.0%)	310 (61.0%)	20 (64.5%)	90 (57.7%)	17 (81.0%)
Age in months	22.0 [12.10, 38.00]	19.5 [11.20, 32.80]	21.0 [12.00; 37.00]	31.0 [21.50;5 0.00]	22.0 [12.0; 40.2]	33.0 [21.0; 52.0]
CAL, *n* (%)	498 (42.30%)	43 (52.4 %)	217 (42.7%)	13 (41.9%)	80 (51.3%)	10 (47.6%)
CRP, mg/L	63.9 [38.10; 100]	95.7 [58.3; 142]	67.1 [37.6; 103]	69.4 [31.70; 105]	67.0 [33.0; 101]	63.0 [22.6; 92.7]
WBC count, ×10^9^/L	14.5 [11.20; 18.40]	14.5 [11.10; 18.10]	14.1 [11.30; 17.90]	12.5 [9.45; 19.10]	14.3 [10.6; 17.7]	12.2 [9.60; 14.0]
NE%	9.33 [6.66; 13.00]	10.4 [8.13; 13.7]	9.22 [6.62; 12.4]	10.3 [5.66; 14.10]	13.1 [7.18; 56.4]	64.3 [44.3; 68.7]
LY%	3.57 [2.31; 5.17]	4.03 [2.05; 6.14]	3.62 [2.37; 5.34]	3.76 [2.14; 5.60]	5.17 [2.65; 19.3]	24.7 [14.0; 42.7]
ESR, mm/h	35.0 [20.0; 50.0]	32.3 [19.00; 50.0]	35.0 [18 ; 50]	39.0 [29.0; 51.50]	35.0 [24.8; 48.5]	40.0 [18.0; 62.0]
Hemoglobin, g/L	111 [104; 118]	107 [95.00; 116]	110 [102; 117]	106 [95.50; 118]	111 [104; 116]	109 [103; 114]
Platelet count, ×10^9^/L	360 [287; 464]	336 [244; 506]	380 [297; 488]	369 [288; 424]	339 [258; 438]	393 [276; 419]
CHO, mmol/L	3.62 [3.15; 4.12]	3.38 [2.87; 4.03]	3.58 [3.08; 4.09]	3.21 [2.78; 3.92]	3.70 (0.81)	3.61 (0.75)
ALB, g/L	38.8 [36.1; 41.1]	37.5 [33.20; 39.70]	38.8 [36.10; 41.00]	35.7 [33.00; 37.40]	40.1 [37.0; 41.7]	38.8 [38.0; 41.2]
AST, U/L	34.4 [26.50; 50.80]	38.5 [27.20; 60.50]	33.2 [26.4; 47.9]	37.6 [29.40; 58.00]	31.4 [25.1; 50.0]	31.7 [27.4; 86.4]
ALT, U/L	25.8 [13.70; 66.50]	25.9 [14.00; 53.20]	26.4 [14.9; 56.1]	38.9 [19.40; 88.10]	21.1 [13.6; 60.4]	59.2 [13.0; 160]
Sodium, mmol/L	135 [133; 137]	135 [133; 137]	136 [133; 138]	134 [130; 138]	135 [133; 137]	135 [133; 137]
CAR	1.69 [1.00; 2.71]	3.43 [2.32; 5.78]	1.79 [0.99; 2.73]	3.42 [1.66; 6.49]	1.58 [0.80; 2.67]	1.60 [0.60; 2.10]
B/A	0.19 [0.09; 0.38]	0.28 [0.16; 0.45]	0.13 [0.09; 0.20]	0.38 [0.14; 0.82]	0.14 [0.09; 0.20]	0.16 [0.13; 0.32]
NLR	2.52 [1.54; 4.56]	3.99 [2.06; 7.10]	2.42 [1.48; 4.23]	5.85 [2.38; 10.10]	2.40 [1.53; 4.90]	2.50 [1.10; 3.50]
TB, μmol/L	5.10 [3.60; 7.90]	7.35 [5.00; 13.60]	5.10 [3.44; 7.73]	11.30 [5.35; 25.10]	5.60 [3.80; 8.43]	6.80 [5.10; 12.7]
PNI	56.10 [49.9; 65.0]	55.2 [46.30; 65.0]	57.1 [50.3; 65.40]	51.7 [40.90; 59.70]	65.8 [52.0; 140]	162 [111; 249]
SII	939 [561; 1,596]	968 [615; 1,667]	942 [568; 1,548]	764 [469; 2,509]	849 [460; 1,572]	783 [463; 1,165]

B/A, bilirubin-to-albumin; ALB, serum albumin; ALT, alanine aminotransferase; AST, aspartate aminotransferase; CAL, coronary artery lesions; CAR, C-reactive protein/albumin ratio; CHO, total cholesterol; CRP, C-reactive protein; ESR, erythrocyte sedimentation rate; ; IVIG, intravenous immunoglobulin; LY%, Percentage of peripheral lymphocyte; ; NE%, Percentage of peripheral neutrophil; NLR, neutrophil to lymphocyte count; PNI, prognostic nutritional index; SII, systemic immune-inflammation index; TB, total bilirubin.

Independent risk factor selection in the training set.

**Figure 2:**
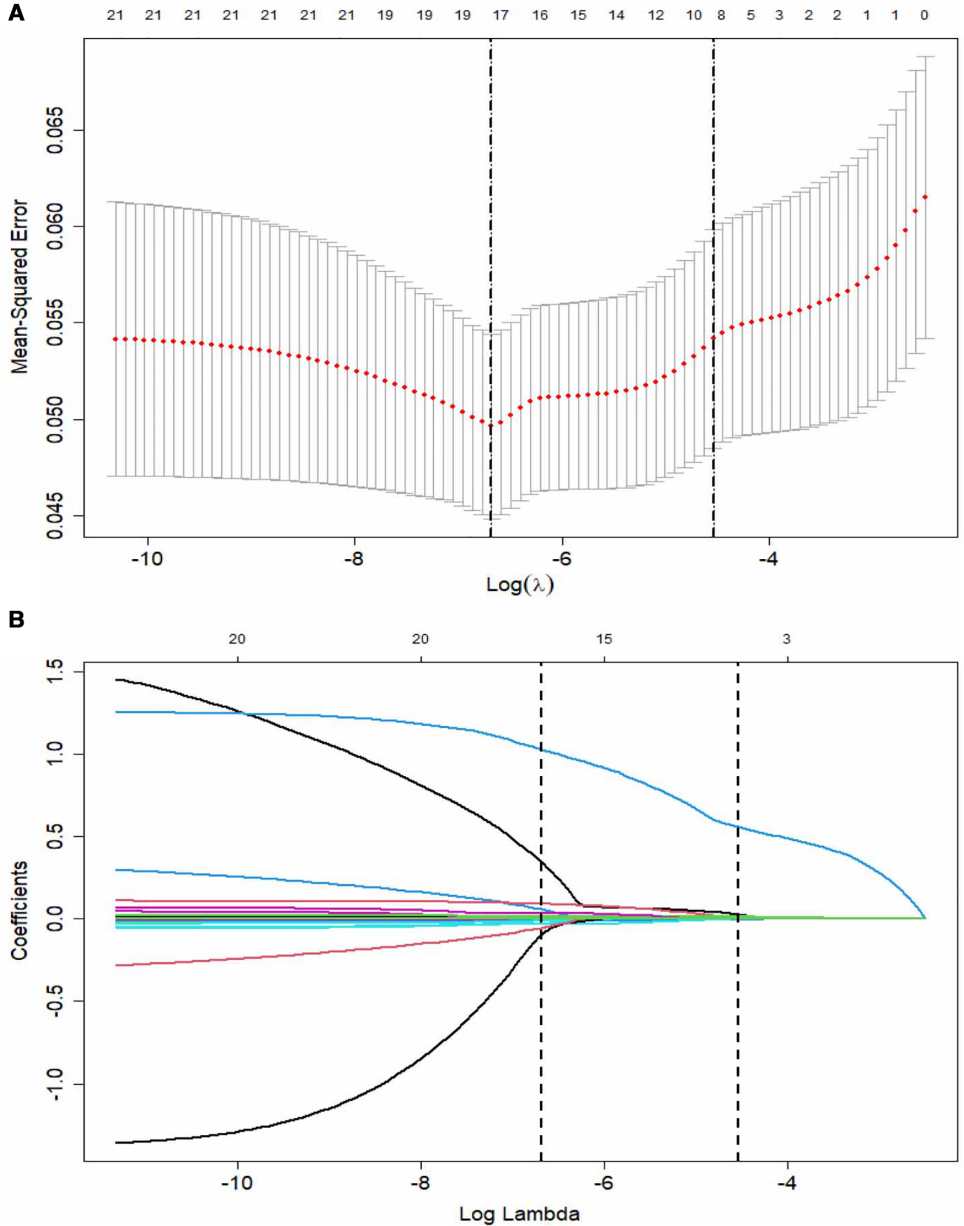
The LASSO logistic regression model for variable selection. (**A**) A coefficient profile plot constructed against the log (lambda) sequence. Dotted vertical lines drawn at the optimal values by using the minimum criteria and the 1 SE of the minimum criteria (the 1-SE criteria). (**B**) Nine variables with nonzero coefficients selected based on the optimal lambda. LASSO, least absolute shrinkage and selection operator; SE, standard error.

LASSO regression was used for the data in the training set, and a total of nine predictors were selected: erythrocyte sedimentation rate, percentage of LY%, CAR, NLR, and levels of AST, sodium, TB, CRP, and Hb (Figure 2).

### Development of a nomogram for IVIG resistance in KD

**Figure 3:**
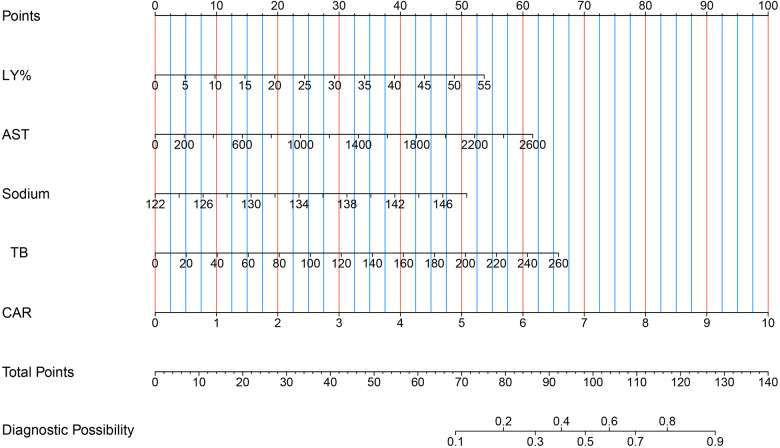
Five risk factors of LY%, AST, sodium, TB and CAR for nomogram prediction model. AST, aspartate aminotransferase; CAR, C-reactive protein/albumin ratio; LY%, lymphocyte percentage; TB, total bilirubin.

First, using the LASSO regression analysis, predictor variables from Table 1 were screened, and then using multivariate logistic regression, the prediction model was established. Five variables that had nonzero coefficients of the LASSO regression model were included in prediction model: LY%, CAR, and levels of AST, sodium, TB (Table 2). These five predictors were used to develop the nomogram (ModA; Figure 3) for predicting the risk probability of IVIG resistance in KD.

**Table 2:** Multivariable logistic regression analyses for prediction after LASSO regression.

Variables	*B*	SE	OR	CI	*p*
LY%	0.104	0.0304	1.109	1.045–1.178	0.001
AST	0.002	0.0007	1.002	1.000–1.003	0.014
Sodium	0.119	0.0389	1.126	1.043–1.215	0.002
TB	0.019	0.0064	1.019	1.007–1.032	0.001
CAR	0.720	0.0735	2.054	1.778–2.372	<0.001

AST, aspartate aminotransferase; CAR, C-reactive protein/albumin ratio; LASSO, least absolute shrinkage and selection operator; LY%, Percentage of peripheral lymphocyte; TB, total bilirubin.

**Figure 4:**
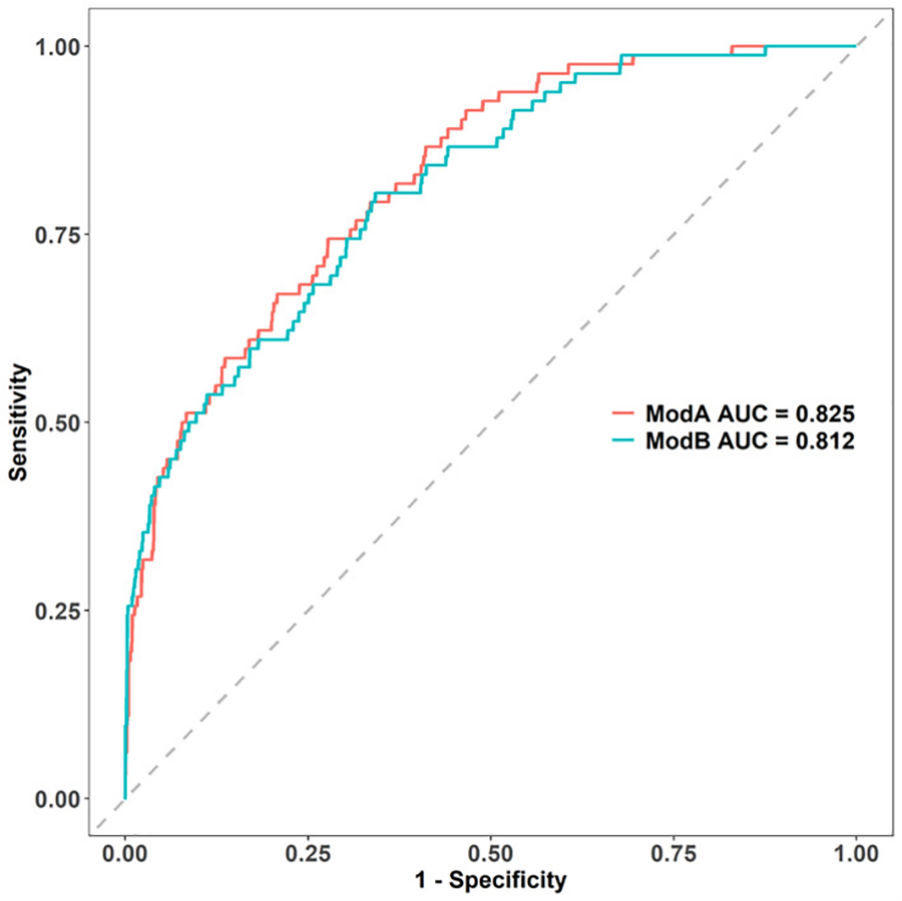
Comparison of AUCs between the two models: modA AUC is 0.826, while modB AUC is 0.812. AUC, area under the curve.

The independent risk factors of IVIG resistance selected in the univariate analysis were analyzed using multivariate logistic regression analysis. Four variables including SII, LY%, and serum TB and CRP levels were used to create the ModB (Supplementary Table S1). The ModA and ModB ROC curves were compared using the DeLong test, but the differences in ROC curves were not statistically significant (*p* = .37; Figure 4). The NRI of ModA was significantly better than that of the ModB (0.304, [95% CI: 0.081–0.527], *p* < 0.05). Therefore, we chose the ModA to predict the IVIG resistance in KD.

### Predictive model validation

**Figure 5:**
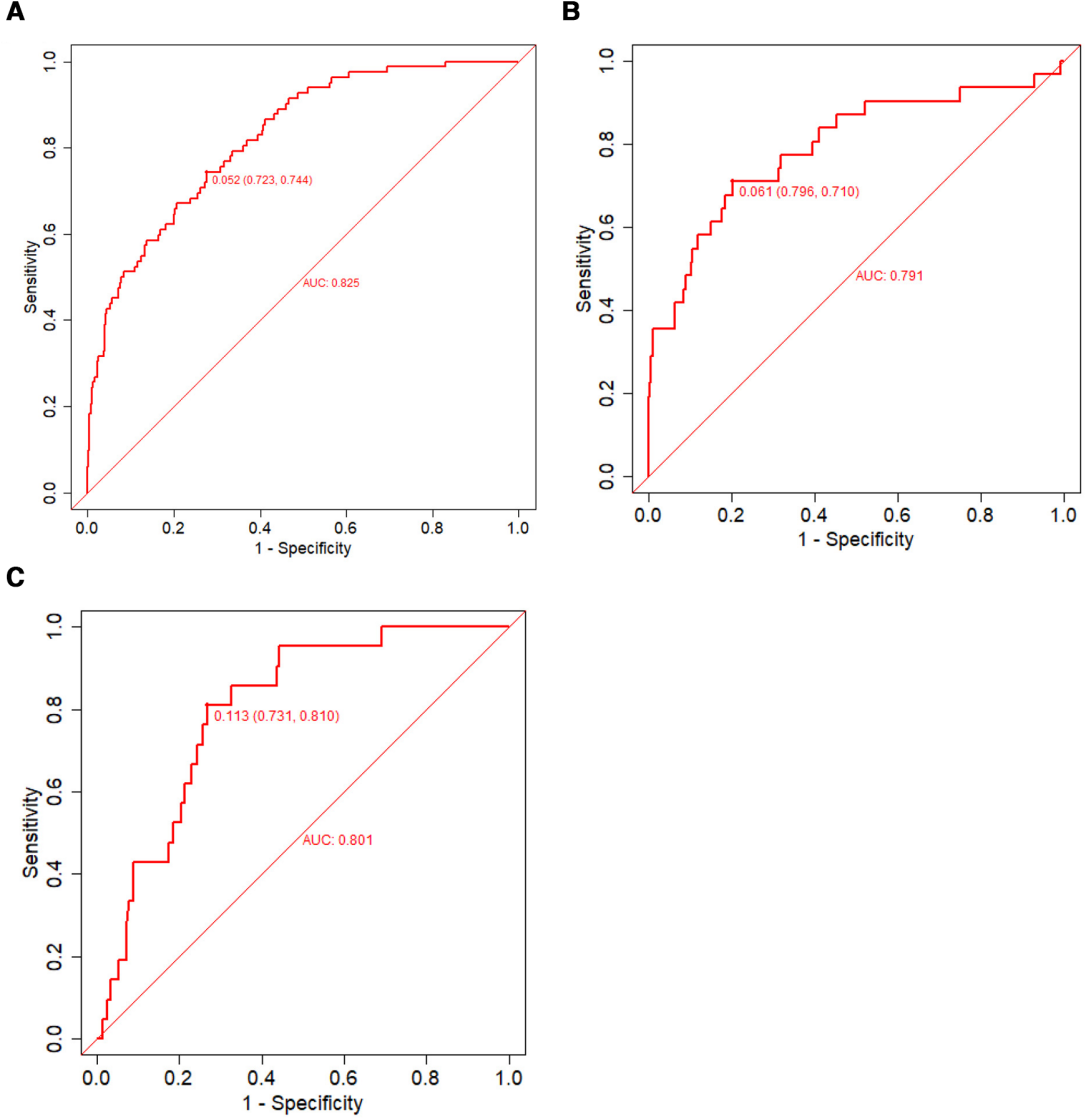
The ROC used to verify the nomogram prediction of IVIG-resistant Kawasaki disease. The *X*- and *Y*-axes represent the false-positive and true-positive rates of risk prediction, respectively. The red broken line represents nomogram performance in the training set (**A**), internal validation set (**B**), and external validation set (**C**). IVIG, intravenous immunoglobulin; ROC, receiver operating characteristic.

Discrimination, a model characteristic, is typically assessed with the area under the ROC (AUC). The AUC of the training set was 0.825 (95% CI: 0.781–0.869), and the sensitivity and specificity were 0.723 and 0.744, respectively. The AUC of the internal validation set was 0.791 (95% CI: 0.694–0.890) and that of the prospective external validation was 0.801 (95% CI: 0.717–0.885; Figures 5A–C).

**Figure 6:**
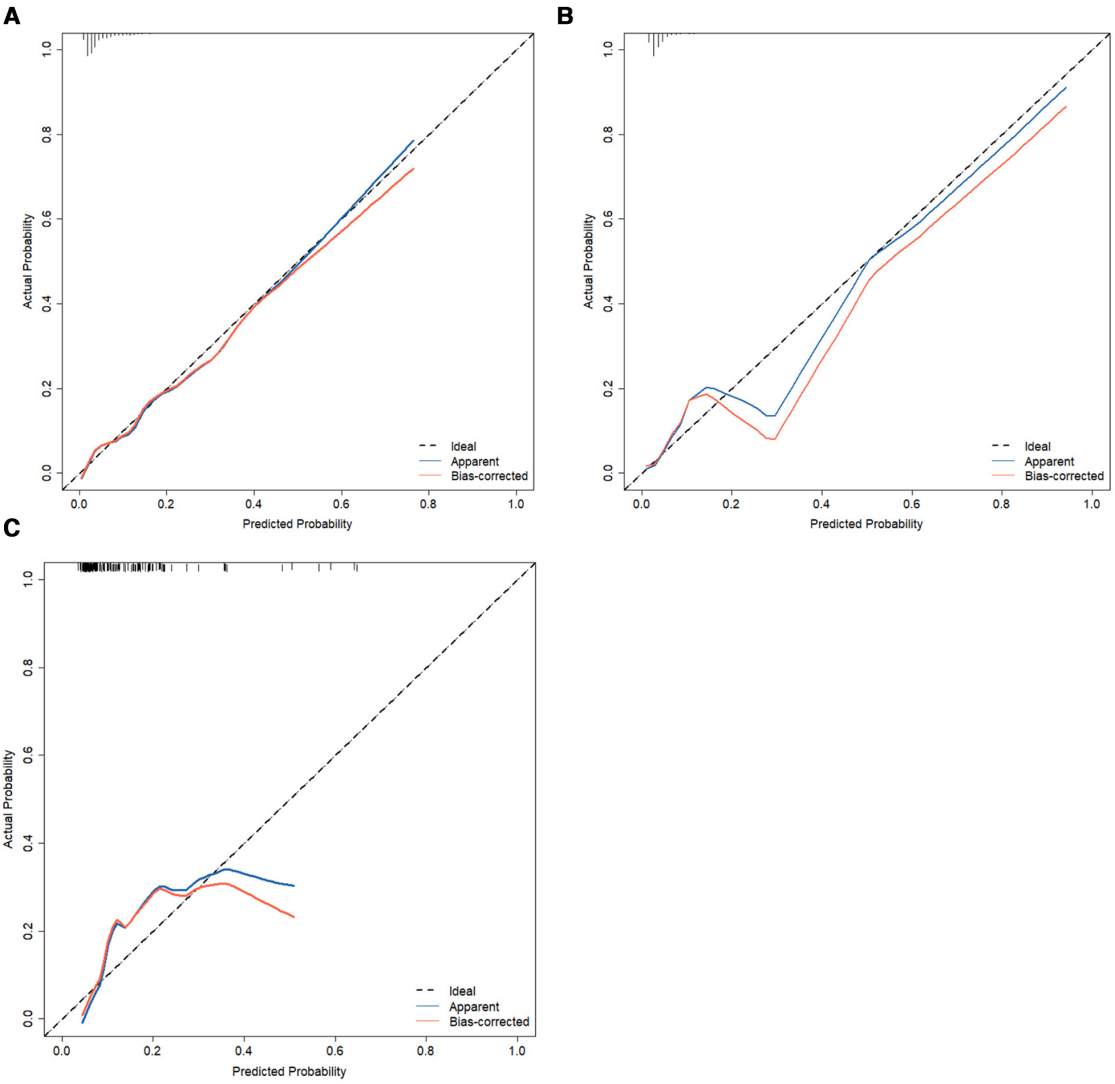
Calibration curves of the nomogram of predictive IVIG-resistant in Kawasaki disease. The diagonal dotted line represents an ideal prediction model, whereas the dashed line represents an ideal reference line, the apparent (blue line) represents the model calibration curve, and the bias-corrected (red line) represents the 500 Bootstrap results. Nomogram performance of the training set (**A**), internal validation set (**B**), and external validation set (**C**). IVIG, intravenous immunoglobulin.

Calibration measurements was performed with the Hosmer–Lemeshow statistical test and calibration plot. The predictive model and the validation set showed a very good degree of fit from the calibration curves. The Hosmer–Lemeshow statistic was 0.42 in the training set, 0.30 in the internal validation set, and 0.41 in the prospective external validation separately (Figures 6A–C).

### Clinical practicality analysis

**Figure 7:**
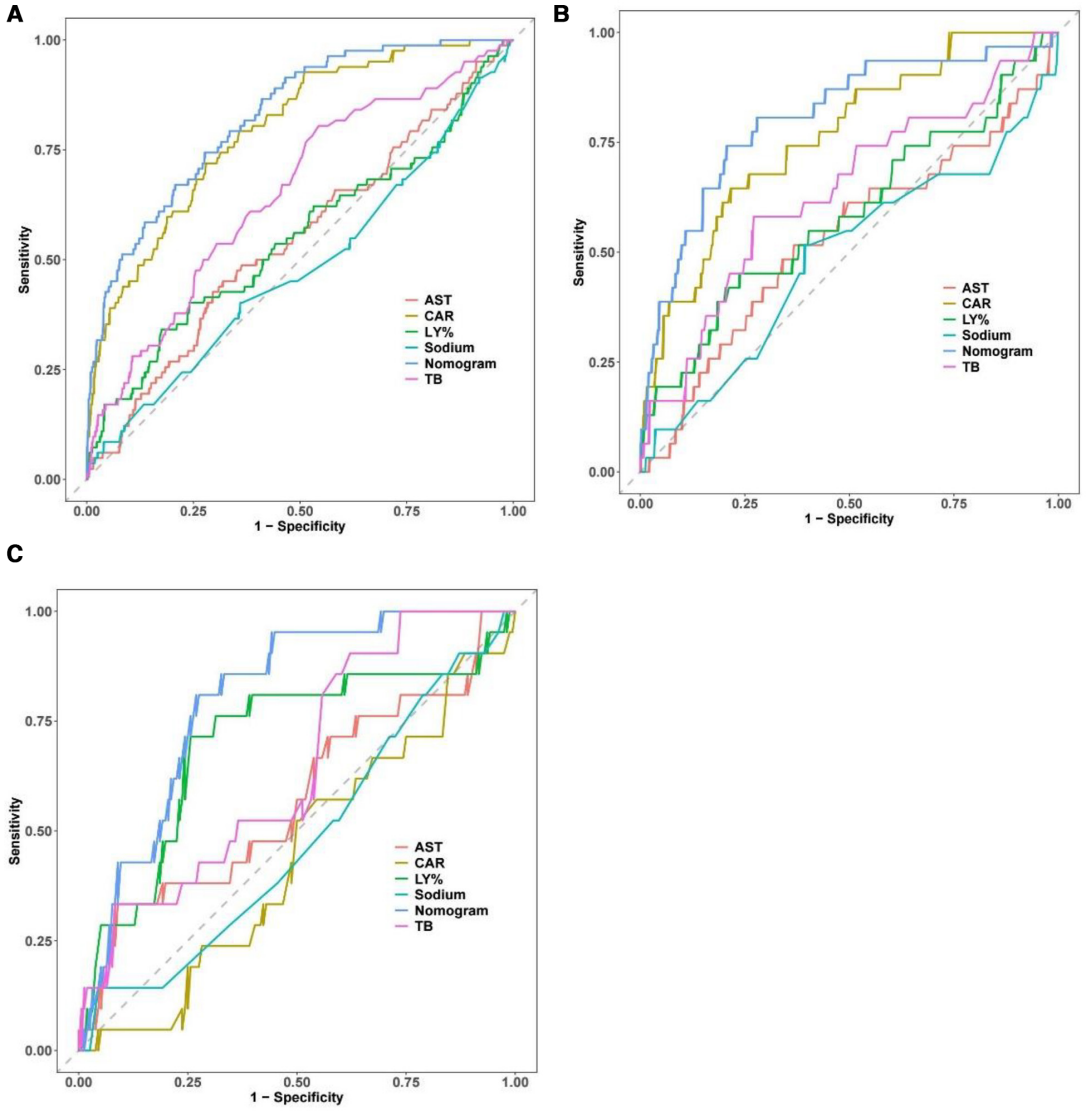
Comparison of the ROC curve between the full dataset of the model including all factors and the model with each factor. The performance of the nomogram is superior to other predictive factors in the training set (**A**) internal validation set (**B**) and external validation set (**C**). ROC, receiver operating characteristic.

**Figure 8:**
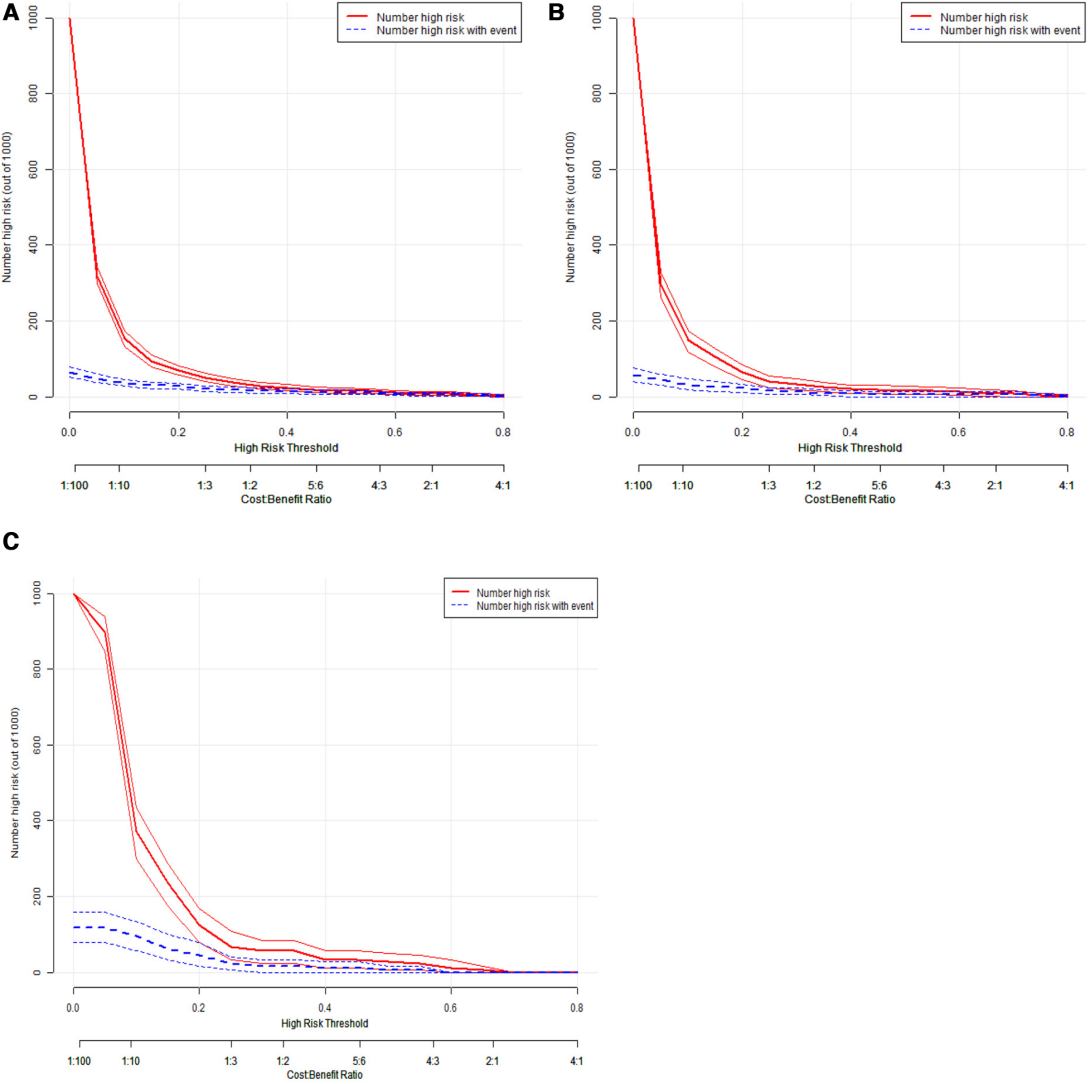
Clinical impact curve of the prediction model for IVIG resistance in Kawasaki disease. The red line represents the number of people judged as high risk by the model at different probability thresholds; The blue line represents the number of true positives judged as high risk by the model at different probability thresholds. The cost benefit ratio represents the proportion of cost and benefit at different probability thresholds. The Y-axis is measured in units of 1,000 people. The figure shows that as the prediction probability increases, the model can predicts that the high-risk population is more closely aligned with the population that actually experiences the outcome event. IVIG: intravenous immunoglobulin.

The AUC of the constructed model was superior to the various model factors in the model (Figure 7). Figure 8 shows the CIC of the predict model and indicates that as the predicted probability increases, the population predicted by the model to have a high risk becomes increasingly consistent with the population of individuals who actually experience the outcome event.

## Discussion

A predictive scoring model was developed and verified internally and externally for early IVIG-resistant KD prediction. The incidence of IVIG non-response from January 2016 to December 2021 was 6.3%, consistent with the results of a previous report (17, 18). The incidence of no response to IVIG increased from January to June 2022 (11.9%), which may be related to the psychological isolation of residents at home during this period. This isolation may have led the patients' parents to avoid hospital visits and may have been the reason patients missed the best time for KD treatment. In our study, we considered LY%, CAR and levels of AST, sodium, and TB as independent risk factors for IVIG resistance in KD.

Although our prediction model reported similar predictors of immunoglobulin resistance in KD to those in previous studies, However, the statistical method used in this study is different from that of previous studies in which only logistic regression analysis was used. Our research method was to first apply LASSO regression to select variables in the data to reduce the dimension of the data and eliminate the collinearity between variables, and then use logistic regression to determine immunoglobulin resistance at an early stage in KD. Our method effectively eliminated data redundancy and improved the accuracy of early prediction. The most important difference between our approach and those of previous studies is that we randomly assigned a large sample population to two groups for modeling and internal validation in a ratio of 7:3 (19). The nomogram was used to present the predictive model to avoid transforming the continuous variables into binary variables and maintain the continuity of the variables. External validation, discrimination, calibration, and clinical rationality tests were performed to verify the accuracy and stability of the model, which provides clinicians with a simple, convenient, and inexpensive early prediction system (20).

Previous studies showed that IVIG treatment before 4 days of onset was an independent risk factor for IVIG resistance. However, the 2017 American Heart Association guidelines recommend that patients who meet the diagnostic criteria should start treatment as soon as possible within 10 days. Early IVIG treatment is beneficial to the prognosis of children with KD. Therefore, we did not consider the duration of IVIG use as a predictor of IVIG resistance (1).

With changes in WBC count, the LY% also changes. The LY% not only reflects the proportion of lymphocytes in WBC counts, but also reflects a much smaller variation in the range of lymphocyte percentage than the absolute value of lymphocytes. Therefore, in this study, we mainly observed and analyzed LY% with a relatively small variation range. We identified increased sodium levels as a predictor of IVIG resistance consistent with the results of Huang et al.'s (18) study. This relative increase in serum sodium levels in IVIG-resistant children may be because of children with IVIG resistance have more severe inflammation and their water intake decreases during the course of the disease. Our study also found that elevated AST level was a risk factor for non-responders to IVIG. AST is the most important enzyme in the liver. When liver cells are injured, cell membrane permeability increases, and AST levels increase owing to its release from the cytoplasm into the blood. AST has been reported in previous KD prediction systems to be associated with IVIG resistance (10, 21, 22), which usually indicates more severe inflammation. AST is also one of the cardiac markers, and an increase in AST level may indicate hypoperfusion caused by cardiac dysfunction. Sano *et al*. found that TB level was a highly significant predictor variable of IVIG non-response, indicating that elevated TB levels were also associated with a higher risk of developing CALs (22). These studies suggest that IVIG resistance is an important indicator of more intense vascular inflammation, which is involved in the injury of hepatocytes and vascular endothelial cells (23). The hepatic reticuloendothelial system is involved in severe liver injury and causes biliary tract inflammation. Some studies have found acute gallbladder swelling on abdominal ultrasound scans in some patients with acute KD that may lead to hyperbilirubinemia.

Our study is the first to include CAR in the prediction model. CAR as a ratio of CRP to ALB was originally proposed by Fairclough (24). In COVID-19 (25), spinal epidural abscess (SEA) (26), transcatheter aortic valve replacement (TAVR) (27), coronary artery ectasia are used to predict systemic inflammatory state and prognosis (28). The CAR has recently been recognized as more useful than CRP or albumin alone for sepsis. Previous study also found that this ratio is associated with the formation of CAL, which is associated with inflammatory processes in children with KD (13). Our study found that CAR was superior to CRP or albumin alone in predicting IVIG resistance in KD, and CRP and albumin are easy to obtain and are routinely tested in the evaluation of KD patients and recommended for clinical use. Serum CRP and albumin levels are commonly associated with IVIG resistance and are included in several risk scoring systems to predict IVIG resistance. CRP, an acute phase reactant protein, is elevated in acute inflammation, tissue injury, and infection, and its levels are positively correlated with the degree of infection. ALB catabolism is directly related to the severity of acute inflammation. Hypoproteinemia, which is common in KD patients, is due to increased permeability and leakiness of vascular inflammation (11, 12, 17). A higher CRP/albumin ratio indicates a higher inflammatory state. Our study found that CRP/albumin ratio was superior to CRP or albumin independently in predicting IVIG resistance in KD, and CRP and albumin are easy to obtain, and are routinely tested in the evaluation of KD patients and recommended for clinical use.

Although standard treatments are available for acute-phase KD, the efficacy of different brands or preparations of IVIG for KD has attracted attention. Suzuki et al. (29) conducted a study of 42,345 KD children who received IVIG treatment. These authors found that 2.8% patients receiving high Na^+^ concentration IVIG as initial treatment developed CAA compared to 3.2% of those who received low concentrations (*p* = 0.031). Suggesting that high-sodium IVIG could effectively reduce the incidence of CALs in KD patients. Other studies have found that IVIG prepared with β-propiolactone may have a higher risk of treatment failure and may increase the duration of antiplatelet or anticoagulant use, and acidification may increase the risk of acute coronary aneurysm (30). These studies suggest that the production process and composition of IVIG products may be potential risk factors for IVIG insensitivity. Because of the retrospective nature of this study, we cannot determine whether these factors contribute to the development of IVIG resistance in children with KD.

The study also has several limitations. First, this study uses a retrospective design to establish the model, because of which we could not explore and determine some relevant factors in depth, such as components of IVIG and potential biomarkers. Second, the results of echocardiography were assessed by different cardiologists, and human error may have been introduced. Nevertheless, based on this large cohort study, reasonable statistical methods, and the results of internal and external validation, we believe that our model can predict IVIG resistance in KD patients in our region.

Early and timely prediction is the premise of individualized treatment of children with IVIG-resistant KD that enables reduction in CAL occurrence and facilitates doctor-patient communication. Identification and prediction of KD IVIG-resistant patients is an urgent clinical need. In our study, LY%; CAR; and levels of AST, sodium, and TB were independent risk factors for IVIG resistance in KD. This information is helpful for clinicians to identify high-risk patients and make clinical decisions at an early stage. CAR may play an important role as an independent predictor of IVIG resistance in KD. More detailed investigation will be performed in a multicenter trial to further analyze factors influencing IVIG components in a larger sample of children with KD.

## Data Availability

The original contributions presented in the study are included in the article/**Supplementary Material**, further inquiries can be directed to the corresponding author.
