# Supplementary material for: A novel model for predicting intravenous immunoglobulin-resistance in Kawasaki disease: a large cohort study
Source: Front Cardiovasc Med. 2023 Jul 28;10:1226592. doi: 10.3389/fcvm.2023.1226592 (PMC10420135; doi:10.3389/fcvm.2023.1226592)
Supplement: Supplementary file 1 [file Datasheet1.pdf]

Supplementary Table 1 Univariable and multivariable logistic regression analyses for prediction of intravenous IVIG non-responders

Variables	Univariable analysis					Multivariable analysis				
	**β**	**SE**	**OR**	**CI**	***P values***	**β**	**SE**	**OR**	**CI**	***P values***
**SII**	0	0.00006	1	1.00-1.00	0.028	0	0.00015	1	0.999-1	0.064
**PNI**	-0.005	0.00743	0.995	0.980-1.009	0.461					
**NLR**	0.07	0.01693	1.072	1.037-1.108	0					
**B/A**	-0.072	0.02635	0.93	0.883-0.979	0.006					
**CAR**	0.646	0.06475	1.908	1.681-2.166	0	0.609	0.06828	1.838	1.608-2.101	0
**TB**	0.031	0.00688	1.031	1.017-1.045	0	0.016	0.00643	1.016	1.004-1.029	0.012
**Sodium**	-0.013	0.01676	0.987	0.955-1.020	0.427					
**AST**	0.001	0.00061	1.001	1-1.002	0.1484					
**ALT**	0.001	0.00059	1.001	0.999-1.002	0.339					
**ALB**	-0.03	0.0134	0.97	0.945-0.996	0.025					
**PLT**	0	0.00075	1	0.998-1.001	0.933					
**Hb**	-0.023	0.01001	0.977	0.945-0.996	0.025					
**LY%**	0.041	0.02983	1.042	0.983-1.104	0.171					
**NE%**	0.023	0.01546	1.023	0.993-1.055	0.141					
**WBC**	0	0.01966	1	0.962-1.039	0.98					
**ESR**	-0.005	0.00511	0.995	0.985-1.005	0.141					
**CRP**	0.013	0.00191	1.013	1.009-1.017	0	-0.017	0.00482	0.983	0.974-0.993	0
**CAL**	0.256	0.22729	1.292	0.828-2.017	0.26					
**Age**	0.004	0.00449	1.004	0.995-1.013	0.345					
**Sex**	0.097	0.23192	1.102	0.699-1.736	0.676					

ALB: serum albumin; ALT: alanine aminotransferase; AST: aspartate aminotransferase; B/A= bilirubin-to-albumin; CAR: C-reactive protein/albumin ratio; CAL: coronary artery lesions; CHO: total cholesterol; CRP: C-reactive protein; ESR: erythrocyte sedimentation rate; LY%: Percentage of peripheral lymphocyte; NE%: Percentage of peripheral neutrophil; NLR: neutrophil to lymphocyte count; OR: odds ratio; PLT: platelet; PNI: prognostic nutritional index; SE: standard error; SII: systemic immune-inflammation index; WBC: white blood cells TB: total bilirubin; 95%CI: 95% confidence interval
